# Nursing Progress and Its Developments

**Published:** 1918-04-13

**Authors:** 


### NURSING PROGRESS AND ITS DEVELOPMENTS.

### XII.—The Local Aims of Local Centres.

It is universally recognised that the College of Nursing must be adequately housed in a central building offering not merely ample accommodation for its varied forms of activity, but calculated in some degree to touch the imagination. As the symbol of the nursing profession its headquarters must be worthy of the great thing it symbolises. It is no less true of the centres now forming in the provinces that their first impulse after launching into a corporate existence turns in the direction of seeking for themselves a local habitation. That is the immediate want which strikes both the outsider, speculating on what the local centres can do for nurses, and the insider possessed of intimate knowledge of nurses' needs and desires. Accordingly, we find that at the first branch meeting at Liverpool, which was fertile in many directions, Miss Cummins was able to announce that their aim was to make their centre a model for other centres to copy, a place to which the majority of nurses working in the county could go without unreasonable expenditure of either time or money.

The aims of every centre must to a certain extent be moulded by the nature of the district it serves. Centres may be formed in localities where comparatively few nurses are working; but under such circumstances no complete organisation can come into being. Such smaller centres may hire a room on certain days in the week, or for certain occasions; may establish a useful bureau of information, open at certain hours; may do good pioneer work for the College; organise popular and valuable meetings for instruction or recreation; and, with but little expenditure of money, may render real support and professional encouragement to all the members within their sphere.

But the larger centres, which can reasonably expect to count their members by hundreds, will inevitably aim at establishing some more permanent and dignified building in which their members may gather. One of their salient purposes will always be the formation of a first-rate club for nurses. In its completest form such a club would occupy premises of some distinction in a central part of the town. It would be run on the lines of a first-class women's club, and many failures have already indicated that such establishments depend for success largely on good domestic management and intelligent catering. The rent and establishment charges would be defrayed by members' subscriptions and by the hire of bedrooms, while the restaurant should be self-supporting. No such enterprise can be run without capital, but there is no reason to doubt that a strong committee commanding the confidence of the members would be able to attract the necessary money for equipment and reserves. The Liverpool Centre has started well, with a strong financial committee, to which outside members of the public have been co-opted, on the ground that "men are much better at getting money than women." In all that benefits nurses the public have a direct interest, and we believe it will not be long before the people of Liverpool have ocular demonstration of the advantage to the town of a strong and united local centre of the College of Nursing.

Liverpool is one of the great ports from which nurses travel to all parts of the Empire, from which nurses from other lands first get a glimpse of the England they have known from childhood in books. Henry James has left on record, in a delightful volume of recollections lately published, his joy on first landing at meeting an English waiter of the good old type in a dingy hotel at Liverpool, and the ecstasy with which he watched him, true to type, place hot water in the slop basin and the muffin dish on top. When the Liverpool local centre is in working order the American nurse who lands from her native shores and sees England first through the mist of a Liverpool afternoon may have her somewhat chilly first impression effaced by the warmth of her reception in a dignified establishment which will typify to her the unity of British nurses. She will feel herself at once to be part of a great corporation restricted within no narrow territorial boundaries, and united by common aims and sympathies.

And if this be true of the strangers who come to our shores, it is equally true of those shy country nurses who seldom meet a comrade and carry on in the remoter parts of the county a work none the less valuable because hidden from view. To the clubhouse they will turn on their rare holidays with certainty of finding something to cheer them professionally, something to uplift, something to instruct. Few guess how bitterly lonely the life of a nurse may be—in the district, at private cases, in the institution. To form a common centre for the help and encouragement of Lancashire nurses, surely this is an aim high enough to inspire the members of the College to a powerful united effort.

